# Vascular Involvement in Behçet’s Disease: Diagnostic Value and Clinical Implications

**DOI:** 10.7759/cureus.92193

**Published:** 2025-09-13

**Authors:** Mouad Faraji, Morad Elaqaoui, Oussama Al Maghraoui, Oualid El Filali

**Affiliations:** 1 Vascular Surgery, Avicenna Military Hospital, Faculty of Medicine of Marrakesh, Caddy Ayad University, Marrakesh, MAR

**Keywords:** angio-behçet, behçet’s disease, classification criteria, diagnosis, vascular involvement

## Abstract

Behçet’s disease is a multisystemic vasculitis diagnosed based on the Classification Criteria for Behçet’s Disease. The asynchronous presentation of symptoms and their classification into major and minor signs can complicate diagnosis, often resulting in delays in diagnosis and treatment. Currently, vascular involvement accounts for only one point in the classification score despite being potentially the most life-threatening manifestation of the disease.

This article presents the case of a young man with a history of recurrent oral ulcers, who was admitted as an emergency for a ruptured popliteal artery aneurysm. The initial evaluation did not meet the diagnostic threshold based on international criteria, thus delaying the initiation of appropriate immunosuppressive therapy. During follow-up, the disease progressed with a new aneurysm in the common femoral artery.

This case advocates for reconsidering the importance of vascular involvement in the diagnostic scoring system for Behçet’s disease, in order to avoid dangerous diagnostic and therapeutic delays in angio-Behçet.

## Introduction

Behçet's disease, first described in 1937 by Turkish dermatologist Hulusi Behçet, is a chronic inflammatory disorder of unknown etiology [1]. Vascular involvement, or angio-Behçet, is a severe and dangerous form of the disease that significantly worsens the prognosis due to the serious complications it causes, as well as the frequent diagnostic delay resulting from the asynchronous onset of the disease’s symptoms, which can make diagnosis more challenging [2].

Despite this clinical reality, the vascular form of the disease (angio-Behçet) is considered a minor criterion (1 point) in the revised International Criteria for Behçet’s Disease (ICBD 2013), whereas manifestations deemed major (oral and genital ulcers or ocular involvement) each count for 2 points. This hierarchy presents a genuine diagnostic challenge: early forms of the disease, particularly those with isolated or asynchronous vascular involvement, may fail to reach the required diagnostic threshold of ≥4 points, leading to delays in initiating appropriate treatment [3].

We present the case of a 34-year-old male patient who arrived at the emergency department at the stage of rupture of a popliteal aneurysm. He was unable to benefit from disease-modifying therapy due to his initially incomplete clinical presentation, which did not meet the threshold of the international classification criteria for Behçet’s disease. Consequently, the patient returned for follow-up with disease progression and a new aneurysmal localization involving the right common femoral artery.

This article aims to re-evaluate the role of vascular involvement among the major classification criteria for Behçet’s disease, in order to address the diagnostic shortcomings of the current criteria and to enable earlier identification of angio-Behçet forms, thereby preventing potentially fatal complications. The ultimate goal is to initiate timely and appropriate long-term treatment to improve the prognosis of this serious condition and to prevent both short- and long-term recurrences.

## Case presentation

The patient was a 34-year-old man with no cardiovascular risk factors, whose medical history was notable for recurrent oral aphthous ulcers. The onset of his condition dates back 20 days, with the appearance of a non-traumatic postero-internal swelling in the lower third of the left thigh, accompanied by severe pain and functional impairment of the ipsilateral lower limb, with no associated symptoms. The condition evolved in an afebrile context with preserved general health status.

Vascular examination upon admission revealed a pulsatile, expanding, painful mass in the postero-internal part of the knee, without signs of limb ischemia. The remainder of the physical examination was unremarkable.

**Figure 1:**
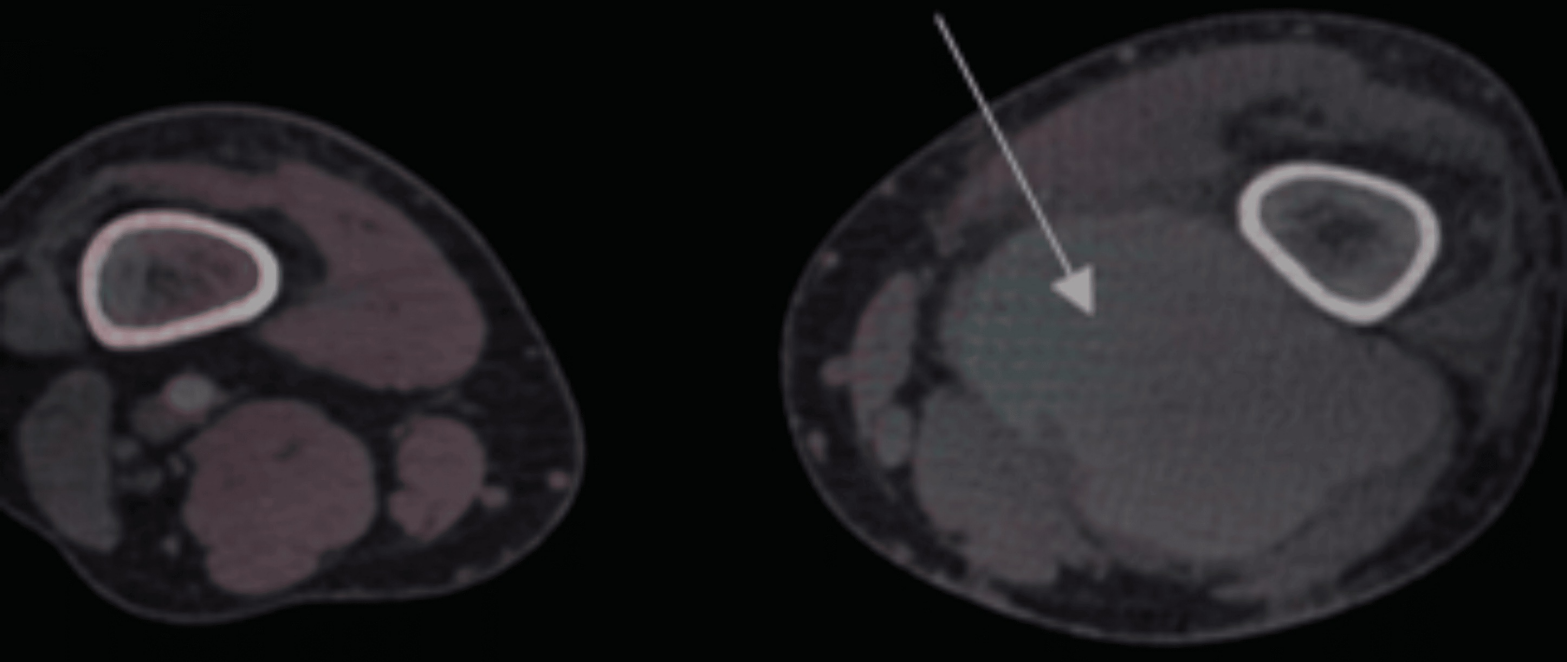
CT angiography of the lower limbs showing a ruptured left popliteal aneurysm. The arrow indicates the ruptured aneurysm.

On admission, laboratory tests were within normal limits with a normal white blood cell count, negative C-reactive protein (CRP) and procalcitonin levels, ruling out sepsis. A CT angiography of the aorta and both lower limbs revealed a fusiform aneurysmal dilation of the left supragenicular popliteal artery, measuring 6.7×4.4×7 cm, with signs of rupture (Figure 1).

**Figure 2:**
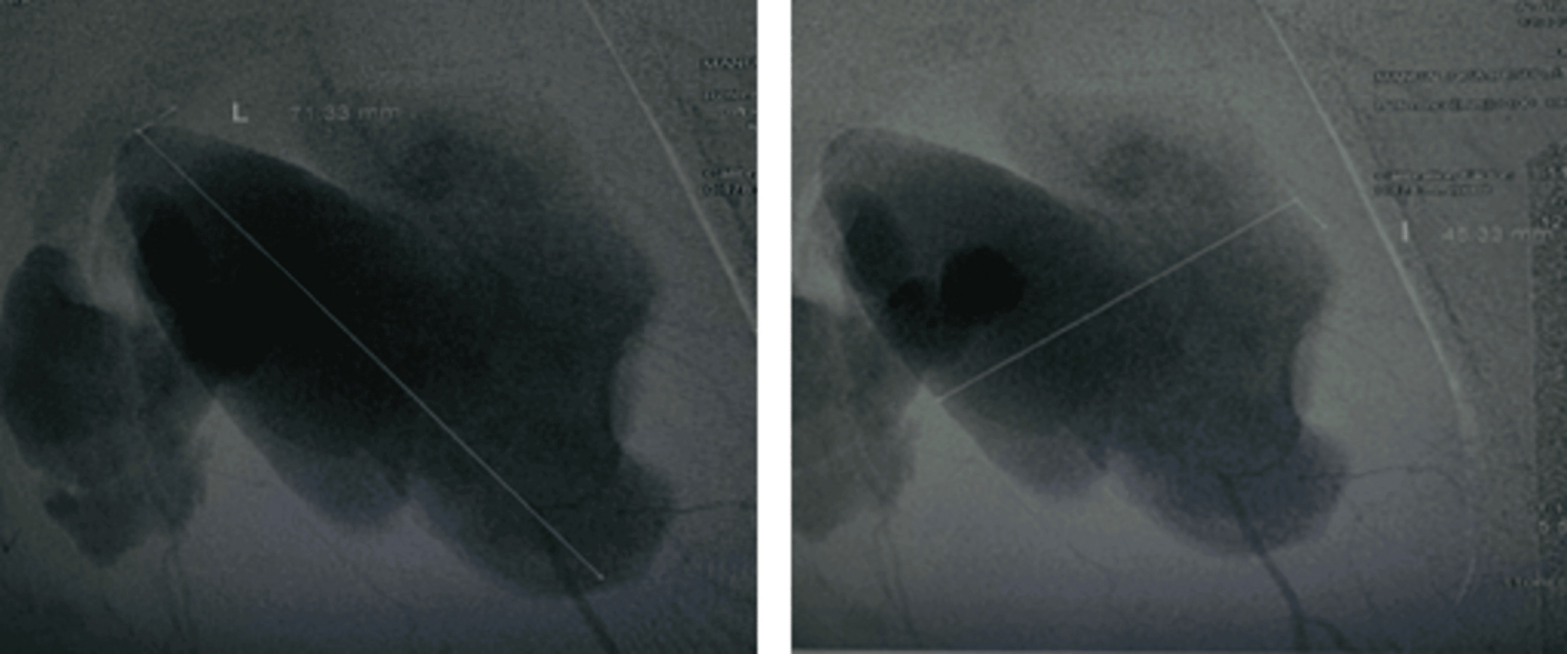
Arteriography of the left lower limb showing a ruptured popliteal aneurysm measuring 71.33×45.33 mm.

Given these findings, arteriography of both lower limbs was performed, confirming a ruptured supragenicular popliteal artery aneurysm, measuring 71.33×45.33 mm, with a poorly perfused and narrowed distal arterial bed (Figure 2).

**Figure 3:**
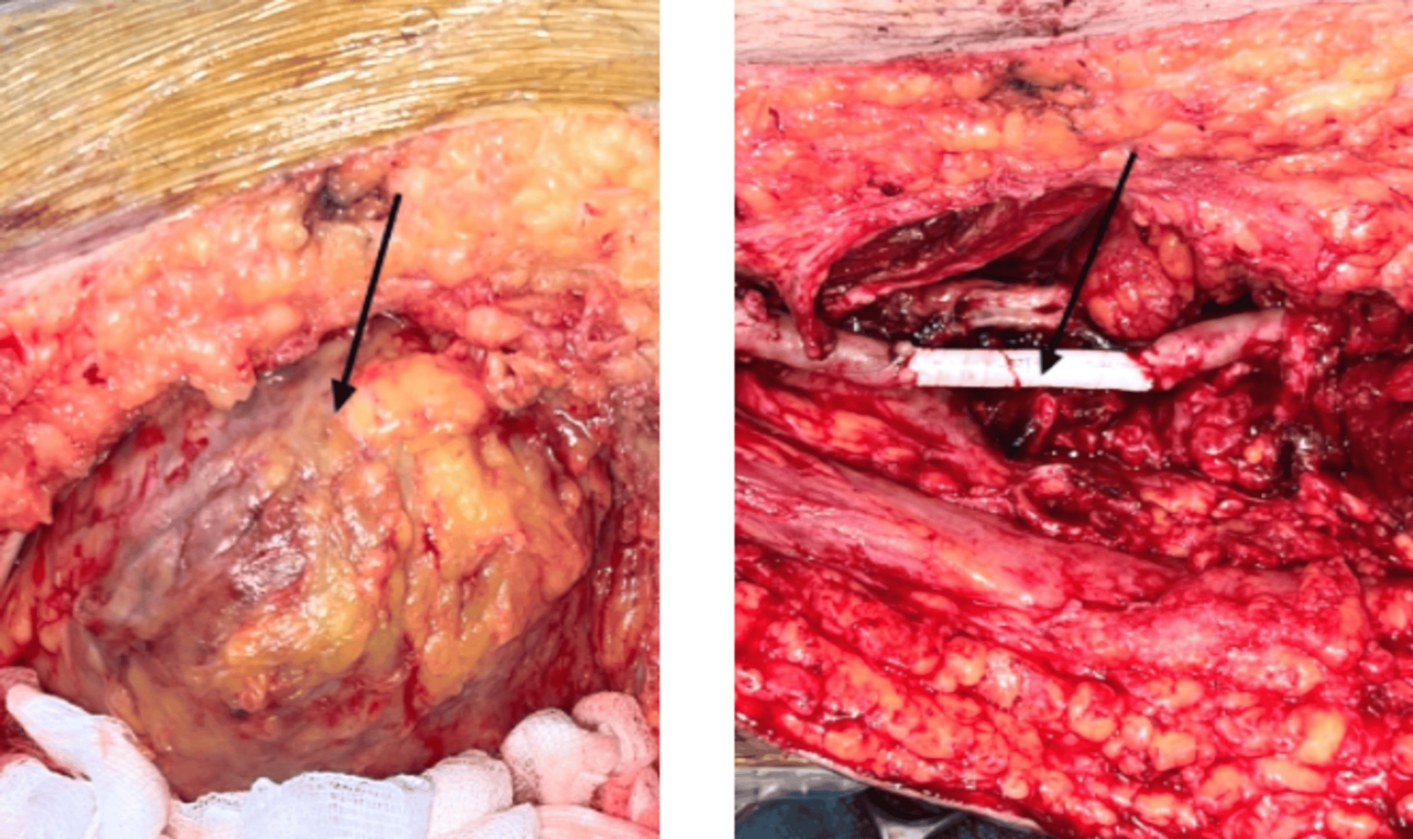
Intraoperative images showing the popliteal aneurysm indicated by the arrow on the left and the prosthetic graft indicated by the arrow on the right.

Emergency surgical intervention was indicated. The patient was transferred urgently to the operating room. Surgical exploration revealed multiple arterial aphthae. The popliteal aneurysm was excluded and a prosthetic graft was placed (Figure 3).

A good pulse was noted at the graft site and distally. Postoperatively, the patient was placed on therapeutic-dose low molecular weight heparin (LMWH). The postoperative course was uneventful, with partial functional recovery of the limb and complete resolution of pain.

An extension workup - including thoracoabdominal CT angiography, ultrasound of the supra-aortic trunks, and transthoracic echocardiography - was unremarkable. At this stage, the patient did not receive immunosuppressive therapy, as his clinical presentation did not fulfill the revised 2013 International Criteria for Behçet’s Disease, showing only vascular involvement and oral aphthous ulcers, without genital ulcers, uveitis, folliculitis, or a positive pathergy test.

Given the suspicion of Behçet’s disease without confirmed diagnosis, the patient was treated with a combination of antiplatelet agents and oral anticoagulants. The clinical course was favorable at the one-month follow-up, with partial recovery of functional capacity in the affected limb. The patient also received follow-up care in internal medicine and neurology.

**Figure 4:**
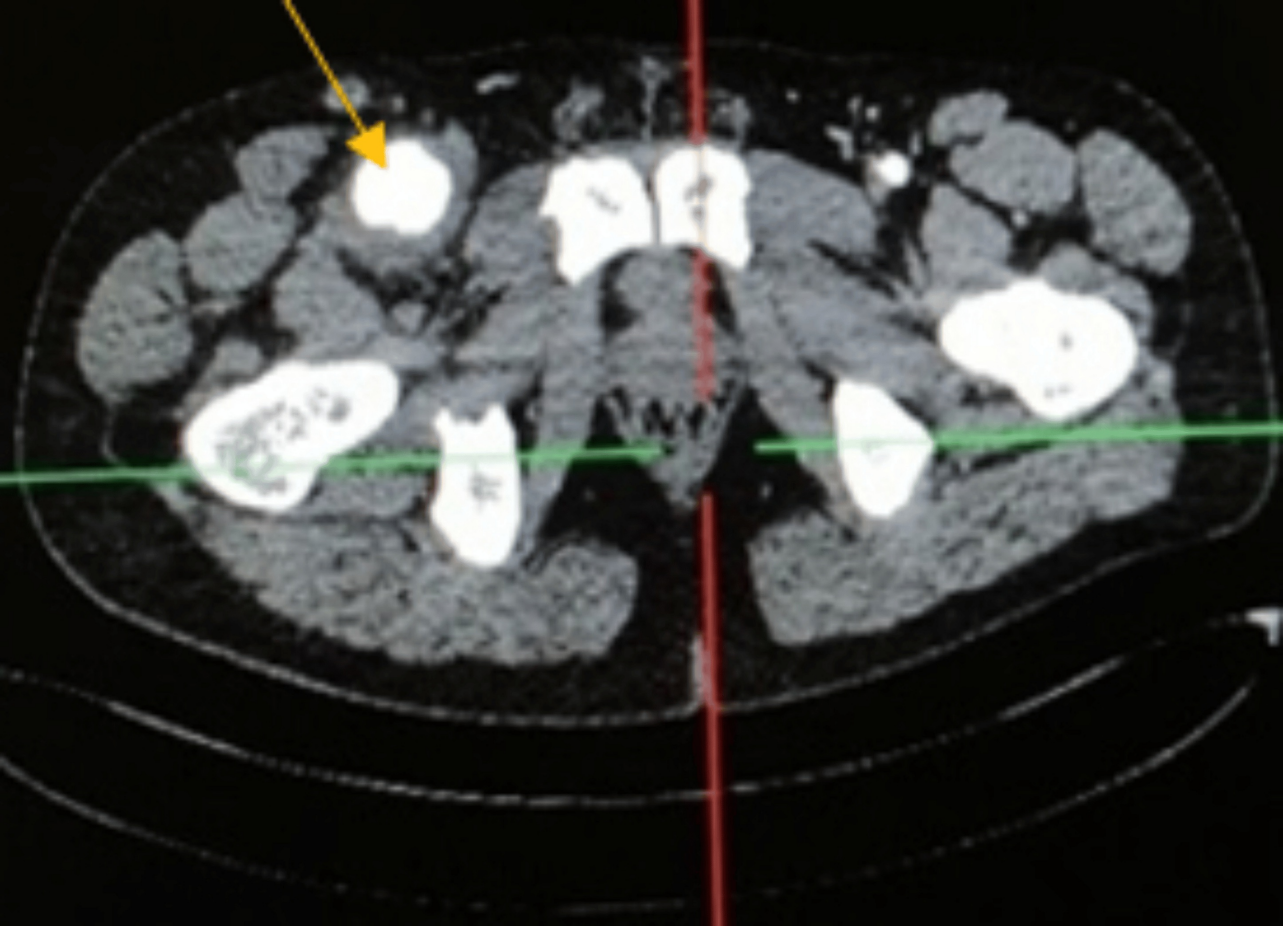
CT angiography of the lower limbs showing an aneurysm of the right common femoral artery as indicated by the arrow.

However, six months later, the patient presented with a painful right inguinal mass. History revealed a recent genital aphthous ulcer. A new CT angiography of the aorta and lower limbs showed a fusiform aneurysmal dilation of the right common femoral artery, measuring 3.6 cm, with circumferential thrombosis not extending to the superficial or deep femoral arteries (Figure 4).

Based on this new presentation, the diagnosis of Behçet’s disease was confirmed using the now completed diagnostic criteria. A covered stent angioplasty was recommended to exclude the femoral aneurysm, and the patient was finally placed on disease-modifying therapy, including corticosteroids and immunosuppressants.

## Discussion

Behçet’s disease is a vasculitis of unknown etiology that predominantly affects young adult men. It typically presents with oral and genital ulcers, often associated with systemic symptoms [1]. The most common manifestations include cutaneous, ocular, and articular involvement, while severe forms may involve the vascular system, heart, central nervous system, and gastrointestinal tract. The disease is ubiquitous, but it mainly affects populations from Central Asia and the Mediterranean basin, following a geographical pattern similar to that of the historic Silk Road [2].

Vascular involvement, also known as angio-Behçet, occurs in approximately 46% of cases, primarily affecting young men [4,5]. Vascular manifestations can be severe and may represent the initial presentation of the disease. Venous thrombosis is the most frequent vascular form, observed in 24.9% to 43% of cases [6,7], while arterial involvement typically presents as aneurysms, described as true “arterial aphthae,” found in up to 16% of patients [5].

The pathogenesis of Behçet’s disease involves non-specific vasculitis that can affect vessels of all sizes and types. Histopathologically, it is a vasculitis of the vasa vasorum, characterized by absence of giant cells, fragmentation and rupture of the media, and immune complex deposition [4,8]. These changes can lead to the formation of true aneurysms or perforations resulting in pseudoaneurysms.

The diagnosis of Behçet's disease remains clinical, based on a set of criteria established by the revised International Criteria for Behçet's Disease (ICBD) from 2013. Oral aphthosis, genital ulcers, and ocular involvement each score two points, while vascular, cutaneous, neurological manifestations, and a positive pathergy test each score one point. A minimum score of four points is required for classification as Behçet's disease. Currently, no specific biological marker exists to support the diagnosis [3].

The main goal in the management of arterial aneurysms is to exclude intra-aneurysmal blood flow to stop progression and prevent rupture, while ensuring adequate distal perfusion. The use of immunosuppressive therapy, particularly corticosteroids in combination with agents like cyclosporine, azathioprine, tumor necrosis factor (TNF) inhibitors, or interferon-α, plays a key role in reducing recurrence risk both in the short and long term [9].

In our case, the patient did not initially receive disease-modifying therapy due to an incomplete clinical presentation that did not meet the diagnostic threshold. This ultimately led to disease progression, highlighting the importance of vascular involvement as a major diagnostic criterion in Behçet’s disease, considering its potentially life-threatening consequences.

## Conclusions

Vascular involvement in Behçet’s disease, particularly arterial manifestations, represents a severe and life-threatening form of the condition. This highlights the need to re-evaluate the diagnostic weight given to vascular features, which are currently underrepresented in the classification criteria for Behçet's disease.

Earlier recognition of angio-Behçet, even in the absence of classic symptoms, is essential to initiate immunosuppressive therapy promptly and prevent serious complications such as aneurysmal rupture or recurrence. This could help reduce the morbidity and mortality associated with this aggressive form of Behçet’s disease.
